# New progress in roles of TGF-β signaling crosstalks in cellular functions, immunity and diseases

**DOI:** 10.1186/s13619-024-00194-x

**Published:** 2024-05-23

**Authors:** Shuchen Gu, Rik Derynck, Ye-Guang Chen, Xin-Hua Feng

**Affiliations:** 1 Center for Life Sciences, Shaoxing Institute, Zhejiang University, Shaoxing, 321000 Zhejiang China; 2 The MOE Key Laboratory of Biosystems Homeostasis & Protection and Zhejiang Provincial Key Laboratory of Cancer Molecular Cell Biology, Life Sciences Institute, Zhejiang University, Hangzhou, 310058 Zhejiang China; 3 Cancer Center, Zhejiang University, Hangzhou, 310058 Zhejiang China; 4 Department of Cell & Tissue Biology, University of California, San Francisco, CA 94143 San Francisco, USA; 5 The MOE Basic Research and Innovation Center for the Targeted Therapeutics of Solid Tumors, Jiangxi Medical College, Nanchang University, Nanchang, 330031 Jiangxi China; 6 The State Key Laboratory of Membrane Biology, Tsinghua-Peking Center for Life Sciences, School of Life Sciences, Tsinghua University, Beijing, Beijing, 100084 China; 7 The Second Affiliated Hospital, Zhejiang University, Hangzhou, Zhejiang China

**Keywords:** TGF-β superfamily, Signaling, Development, Immunity, Diseases

## Abstract

The family of secreted dimeric proteins known as the Transforming Growth Factor-β (TGF-β) family plays a critical role in facilitating intercellular communication within multicellular animals. A recent symposium on TGF-β Biology - Signaling, Development, and Diseases, held on December 19–21, 2023, in Hangzhou, China, showcased some latest advances in our understanding TGF-β biology and also served as an important forum for scientific collaboration and exchange of ideas. More than twenty presentations and discussions at the symposium delved into the intricate mechanisms of TGF-β superfamily signaling pathways, their roles in normal development and immunity, and the pathological conditions associated with pathway dysregulation.

## Main text

### Introduction

TGF-β family signaling pathway plays a crucial role in regulating vital physiological processes in multicellular organisms, facilitating essential communication and coordination among cells, tissues, and organs throughout an organism’s lifespan. The TGF-β family encompasses three subfamilies: Activin, Bone Morphogenetic Protein (BMP), and TGF-β, with a total of 33 genes encoding distinct family proteins in humans (for reviews, see Morikawa et al. 2016 and Tzavlaki and Moustakas 2020). Homodimeric and heterodimeric molecules intricately control a wide range of cell functions from regulating diverse signaling pathways, nuclear epigenetic regulation, gene expression and mRNA processing, secretion of extracellular cytokines and bioactive molecules, and reprogramming extracellular matrix protein expression and homeostasis. Context-dependent regulation, depending on factors such as cell type, developmental stage, and spatial cues, is essential for ensuring the physiological functions. An imbalance in the TGF-β signaling significantly influences the development of fibrosis by enhancing the extracellular matrix deposition. Additionally, TGF-β plays a role in advancing tumors by promoting epithelial to mesenchymal differentiation with changes in cell behavior, suppressing immune responses, and stimulating the neovascularization during the later stages of cancer. Moreover, disturbances in the TGF-β signaling pathway are linked to a range of human pathological conditions such as anemia, inflammatory diseases, impaired wound healing, and cardiovascular diseases. Their physiological responses and pathological dysregulation underscore the extensive control by TGF-β family proteins of the regulation of bodily functions and the development of diseases.

Even after more than forty years of intense research, new aspects of TGF-β activity are still being uncovered. The recent symposium on “TGF-β Biology-Signaling, Development and Diseases”, sponsored by the Life Sciences Institute of Zhejiang University and the Chinese Society of Cell Signaling, was held at Thousands Islands Lake, Hangzhou in China. The conference brought together a group of scholars from within the country and around the world, to discuss the latest developments in our understanding of TGF-β signaling pathways. Key messages and recommendations emerging from these presentations and discussions are summarized in this report.

### TGF-β signaling and cancer

The role of TGF-β in cancer is multifaceted, encompassing both inhibitory and promoting effects that vary depending on the stage of the disease. Consequently, the development of TGF-β-based treatments has proven to be difficult due to the potential for systemic cytotoxicity. Rik Derynck (University of California - San Francisco, USA) discussed the pivotal roles of TGF-β signaling in cancer progression. He explained how TGF-β promotes the invasion and spread of cancer cells, as well as the development of stem cell characteristics, contributing to resistance to therapy. Derynck also gave an overview of clinical trials that are currently in progress for drugs targeting TGF-β. He highlighted the complex interaction between TGF-β released by cancer cells and other elements in the tumor microenvironment (TME), emphasizing the decisive role of TGF-β in cancer advancement (Derynck et al. 2021). Selective targeting specific integrins, which are required for TGF-β activation, can promote T cell infiltration and immune–activated TME, thereby repressing cancer progression in model systems. Repression of TGF-β signaling is seen as an essential and prime approach to improve the effectiveness of immunotherapies, even for tumors with cancer cells that do not respond to TGF-β (Derynck et al. 2021).

Carl-Henrik Heldin from Uppsala University (Sweden) presented findings on the development of selective TGF-β inhibitors targeting only the tumor-promoting effects of TGF-β. His research uncovered mechanisms that trigger invasiveness and metastasis. Activation of p38 MAP-kinase and PI3-kinase pathways occurs independently of the kinase activities of the TGF-β receptors, relying instead on the ubiquitin ligase TRAF6, which binds to TβRI, the type I TGF-β receptor (Hamidi et al. 2017). Conversely, activation of Src relies on the phosphorylation of a tyrosine residue in TβRI by TβRII, the type II TGF-β receptor, creating a docking site for the SH2 domain of Src and exposing the catalytic pocket of its kinase domain (Yakymovych et al. 2022). These unique regulators offer the potential to selectively inhibit tumor-promoting pathways while preserving the tumor-suppressing effects, holding promise for the treatment of advanced cancers. Ying Zhang’s studies at National Institutes of Health (USA) proposed that the presence or absence of Smad4 may dictate the outcome of TGF-β signaling toward tumor progression. The deletion of Smad4 may not just initiate tumors, but could also act as a factor that promotes tumor metastasis in gastrointestinal cancers. When Smad4 is absent, Smad3 is available to engage in regulation of alternative splicing that promotes epithelial-to-mesenchymal transition, metastatic dissemination and cancer drug resistance (Tripathi et al. 2019). It would be interesting to investigate whether Smad3 needs the epigenetic regulator TRIM33 or related proteins to mediate the response.

Signaling by the TGF-β family results in epigenetic changes and changes in gene expression. Qiaoran Xi from Tsinghua University in China presented her research on understanding BMP signaling in diffuse intrinsic pontine glioma (DIPG). She examined the context-dependent features of the BMP signaling pathway in DIPG subtypes with different onco-histone variants such as H3.3K27M or H3.1K27M (Sun et al. 2022). These findings offer promise for developing preclinical therapeutic strategies for DIPG. In addition, her research group concentrates on the H3.3K27M DIPG subtype, delving into the oncohistone-driven CREB5/ID1 gene expression axis to elucidate its role in promoting malignancy.

Three investigators from the Life Sciences Institute (LSI) of Zhejiang University shared their TGF-β-related studies in cancer. Long Zhang presented findings that breast cancer cells transport active TGF-β type II receptor (TβRII) via exosomes to recipient cells, thus promoting epithelial-mesenchymal transition in tumor cells and enhancing CD8 + T cell exhaustion (Xie et al. 2022). Jianping Jin, in collaboration with Xin-Hua Feng, identified the autophagy regulator AMBRA1 as a ubiquitin ligase for non-proteolytic modification of Smad4 to promote TGFβ-induced metastasis in breast cancer (Liu et al. 2021). Mu Xiao from Xin-Hua Feng’s group reported the RNA-binding protein SFPQ, which is often upregulated in cancers, as a novel yet potent suppressor of TGF-β signaling. SFPQ excluded Smad4 from the Smad complex and chromatin occupancy through phase separation. Loss of SFPQ phase separation activities rendered human cells hypersensitive to TGF-β responses (Xiao et al. 2024). Additional RNA-binding proteins can have various effects on TGF-β responses through different mechanisms (unpublished data).

### TGF-β signaling and immunity

TGF-β plays a crucial role in governing the growth, activation, proliferation, differentiation, and apoptosis of immune cells. TGF-β is instrumental in maintaining quiescence and controlling the activation of naive T cells within CD4 + T cells, inhibits differentiation and function of Th1 and Th2 cells while promoting the differentiation of Th17, Th9 cells and regulatory T cells (Tregs). Investigating the role of TGF-β in T cells, Chen Dong at Westlake University (China) highlighted TGF-β’s role in controlling the stem-like population of CD8 + T cells. Notably, the selective expression of the transcription factor Bcl6 in stem-like CD8 + T cells is intricately regulated by TGF-β via Smad2 (Sun et al. 2023). This control serves to suppress the IL-2/STAT5 pathway, offering insights into the functional interplay between TGF-β and Bcl6 in cancer immunity, as well as providing potential targets for cancer immunotherapy. WanJun Chen from National Institutes of Health (USA) discussed his work on TGF-β regulation of Treg cells and T cell quiescence, as well as Th9 cells in autoimmunity and cancer. TGF-β is essential for inducing Foxp3 expression in naive T cells, which leads to the development of Tregs. Moreover, aside from its effect on CD4 + T cells, TGF-β also extends its regulatory influence to innate lymphoid cells and gut intraepithelial lymphocytes. Lastly, Jing Li from the University of Pittsburgh (USA) described the role of upregulation of TGF-β receptors in the regulation of KIR + CD8 + T cells in autoimmune diseases (Li et al. 2022).

The TGF-β signaling pathway interacts with other pathways related to the immune system. Bing Su from Shanghai Jiao Tong University/Shanghai Institute of Immunology (China) demonstrated the effects of MEKK2 and MEKK3 on the functionality of blood vessels, signaling of innate immune receptors, as well as the development and functioning of T cells. Importantly, he discovered substantial communication between the MAPK pathways downstream of MEKK2 and MEKK3 and TGF-β signals, which impact the differentiation of T cell differentiation (Chang et al. 2011) and the stability of endothelial cells (Deng et al. 2021). These findings reveal cooperation between the MAPK pathway with TGF-β signals in various cell types to control immune and blood function. Yi Yu from Xin-Hua Feng’s group from the LSI described the noncanonical functions of Smad7 in STAT3 activation (Yu et al. 2017), which were strengthened by PRMT5-mediated methylation of Smad7 (Cai et al. 2021). Furthermore, Smad7 deficiency significantly promotes tumor growth by impairing transcription of *CIITA* (encoding Class II MHC transactivator) in dendritic cells (unpublished data).

Li Yang from the National Institutes of Health (USA) found that specifically eliminating the *Tgfbr2* gene, encoding TβRII, in myeloid cells led to enhanced tumor dormancy and prevented tumor metastasis (Pang et al. 2013; Ishii et al. 2018; unpublished data). Notably, dampened TGF-β signaling in myeloid cells caused type 1 inflammation, leading to cerebral vasculitis and spontaneous stroke. This research emphasizes the vital importance of myeloid-specific TGF-β signaling in cancer immune evasion, and is significant in its provision of a valuable animal model for stroke.

Li Yu from Tsinghua University in China made an intriguing discovery of a new cellular structures known as migrasomes (Ma et al. 2015). Migrasomes, which range in size from approximately 500 to 3000 nm, are generated by migrating cells, carry cargos, and mediate intercellular communication. In addition to this original discovery, Yu also reported an unexpected finding regarding the involvement of TGF-β in regulating the biogenesis of migrasomes (unpublished data). This suggests that TGF-β may play a role in mediating certain physiological processes through the formation and function of migrasomes. These findings open up new possibilities for understanding the mechanisms underlying the diverse physiological effects of TGF-β.

### Signaling crosstalk in development and diseases

The TGF-β superfamily plays a key role in multiple facets of development and maintaining tissue balance, frequently interacting with other pathways such as NF-κB, Wnt and Hippo pathways. Ye-Guang Chen (Nanchang University/Tsinghua University, China) discovered that maintenance of intestinal epithelial homeostasis is highly dependent on BMP signaling, which restricts the stem cell characteristics of Lgr5 + cells (Qi et al. 2017). Additionally, BMP signaling is crucial for regulating the equilibrium of intestinal stromal cells and their impact on epithelial function. Disruption of BMP signaling in Gli1^+^ stromal cells leads to an expanded stromal cell population, resulting in mucosal inflammation and the formation of colon polyps. The increased secretion of interleukins, particularly IL-1 and IL-17α, by stromal cells intensifies mucin production by goblet cells through NF-κB signaling, leading to structural changes, epithelial barrier disruption, and the progression of polyps (Wang et al. 2023b). This discussion has provided an in-depth understanding of the intricate involvement of BMP signaling in intestinal stromal cells and its consequences for epithelial function. Seong-Jin Kim, from the GILO Foundation Research Institute in Korea, discussed how microtubule associated serine/threonine kinase family member 4 (Mast4) regulated the fates of mesenchymal stem cells (MSCs). While Mast4 promotes osteogenesis by facilitating Wnt signaling, suppression of Mast4 by TGF-β blocked Sox9 degradation to enhance chondrogenesis. Mast4 depletion in MSCs favors cartilage formation and regeneration, highlighting its essential role in determining MSC fate development into cartilage or bone (Kim et al. 2022).

TGF-β and related cytokines control various aspects of life as morphogens, from embryogenesis to adult organ development and functions. During embryogenesis, different members may even have opposing roles. Duanqing Pei (Westlake University, China) explored cell fate decision processes by delving into the networks of how morphogens impact transcription factors and chromatin modifiers. While the pluripotency factor Sall4 collaborates with the NuRD complex to specify pluripotent cell fate (Wang et al. 2023a), BMP4 promotes the dissociation of Sall4 from NuRD and orchestrates a gene regulatory network towards primitive endoderm (unpublished data). In the zebrafish model, Nodal acts as a head organizer, while BMP signaling acts as a caudal organizer (Xu et al. 2014). Pengfei Xu (Zhejiang University, China) examined the role of Nodal and BMP signaling in coordinating the formation and arrangement of various tissues and organs in early embryo development.

A few presentations focus on investigating the signaling components of the Hippo pathway known to interact with the TGF-β pathway, which is vital for understanding their involvement in development and cancer. Kunxin Luo (University of California – Berkeley, USA) showed that pathway-specific transcription factors like TAZ utilize phase separation to efficiently activate transcription. Subcellular condensates can function as scaffolds to concentrate proteins with similar functions or to insulate protein complexes, generating specificity in different signaling pathways (Lu et al. 2020). Xiaohua Yan, at Nanchang University in China, brought attention to the contextual regulatory mechanisms that control the crosstalk between Smad2 and Smad3, and TEAD4 in the progression of liver cancer. Jianping Jin discovered that the understudied ubiquitin ligase RNF214 acts as a non-proteolytic polyubiquitin ligase on TEAD, thus amplifying the transcriptional capabilities of YAP/TAZ-TEAD in liver cancer. Lastly, the cGAS-STING pathway was the subject of discussion, as Pinglong Xu of the LSI presented new findings on the involvement of cGAS-STING in fibrosis. His presentation also underscored the influence of a viral protein on TGF-β signaling in liver cirrhosis related to hepatitis (Zhang et al. 2022).

### Perspectives

The 2023 Symposium on TGF-β Biology-Signaling, Development, and Diseases brought together a diverse group of scientists specializing in various aspects of TGF-β research. The insights exchanged advanced our understanding of this intricate TGF-β signaling pathway, particularly in terms of strategies for regulating TGF-β signaling and its pivotal role in shaping an immune-suppressing microenvironment. These mechanisms have emerged as a focal point for augmenting the effectiveness of current and future immunotherapies, particularly in tumors with limited TGF-β responsiveness. The symposium sparked discussions on potential avenues for treatment, emphasizing a shared belief in reducing TGF-β signaling to improve outcomes in cancer immunotherapies. Attendees explored potential partnerships in research endeavors and clinical trials, underscoring the importance of inhibiting TGF-β signaling as a therapeutic approach in cancer treatment.

## Data Availability

Not applicable.

## Abbreviations

AMBRA1: Autophagy and beclin 1 regulator 1
BMP: Bone Morphogenetic Protein
CD4: Cluster of differentiation 4
CD8: Cluster of differentiation 8
cGAS: cGAS cyclic GMP-AMP synthase
CREB: cAMP responsive element-binding protein
DIPG: Diffuse intrinsic pontine glioma
ID1: Inhibitor of DNA binding 1
IL2: Interleukin 2
KIR: Killer cell immunoglobulin like receptor
Lgr5: Leucine rich repeat containing G protein-coupled receptor 5
MAPK: Mitogen activated protein kinase
Mast4: Microtubule associated serine/threonine kinase family member 4
MEKK: MAPK/Erk kinase kinase
MHC: Major histocompatibility complex
MSC: Mesenchymal stem cell
NF-κB: Nuclear factor κ B
NuRD: Nucleosome remodeling and deacetylase complex
PRMT5: Protein arginine methyltransferase 5
SFPQ: Splicing factor proline and glutamine rich
Src: Rous sarcoma oncogene cellular homolog
STAT: Signal transducer and activator of transcription
STING: Stimulator of interferon response cGAMP interactor 1
TAZ: WW domain containing transcription regulator 1
TβRI: Transforming Growth Factor-beta type I receptor
TβRII: Transforming Growth Factor-beta type II receptor
TEAD: TEA domain transcription factor 1
TGF-β: Transforming Growth Factor-beta
TME: Tumor microenvironment
TRAF6: TNF receptor associated factor 6
TRIM33: Tripartite motif containing 33
YAP: Yes-associated protein
